# HER2/PI3K/AKT pathway in HER2-positive breast cancer: A review

**DOI:** 10.1097/MD.0000000000038508

**Published:** 2024-06-14

**Authors:** Linghui Pan, Jinling Li, Qi Xu, Zili Gao, Mao Yang, Xiaoping Wu, Xuesen Li

**Affiliations:** a Institute for Cancer Medicine and School of Basic Medical Sciences, Southwest Medical University, Luzhou, China; b Department of Laboratory Medicine, Chonggang General Hospital, Chongqing, China.

**Keywords:** HER2, HER2-AKT pathway, HER2-positive breast cancer, trastuzumab resistance

## Abstract

Breast cancer is currently the most commonly occurring cancer globally. Among breast cancer cases, the human epidermal growth factor receptor 2 (HER2)-positive breast cancer accounts for 15% to 20% and is a crucial focus in the treatment of breast cancer. Common HER2-targeted drugs approved for treating early and/or advanced breast cancer include trastuzumab and pertuzumab, which effectively improve patient prognosis. However, despite treatment, most patients with terminal HER2-positive breast cancer ultimately suffer death from the disease due to primary or acquired drug resistance. The prevalence of aberrantly activated the protein kinase B (AKT) signaling in HER2-positive breast cancer was already observed in previous studies. It is well known that p-AKT expression is linked to an unfavorable prognosis, and the phosphatidylinositol-3-kinase (PI3K)/AKT pathway, as the most common mutated pathway in breast cancer, plays a major role in the mechanism of drug resistance. Therefore, in the current review, we summarize the molecular alterations present in HER2-positive breast cancer, elucidate the relationships between HER2 overexpression and alterations in the PI3K/AKT signaling pathway and the pathways of the alterations in breast cancer, and summarize the resistant mechanism of drugs targeting the HER2–AKT pathway, which will provide an adjunctive therapeutic rationale for subsequent resistance to directed therapy in the future.

## 1. Introduction

According to GLOBOCAN2020 data report by the Global Cancer Observatory of the International Agency for Research on Cancer, breast cancer emerged as the predominant cancer globally among women in the year 2022. The report revealed a staggering 226 million cases of breast cancer, comprising 11.7% of all cancer cases. Furthermore, it stood as the primary cause of female mortality, accounting for 15.5% of all cancer-related deaths.^[1]^ Breast cancer encompasses a diverse array of malignancies with varying characteristics, usually considered to consist of at least 4 different clinically relevant molecular subtypes: luminal A type (ER + and/or PR+/HER2-), luminal B type (ER + and/or PR+/HER2+), HER2-positive (ER-/PR-/HER2+), and basal-like subtype (ER-/PR-/HER2-), of which HER2-positive breast cancer represents 15% to 20% of all breast cancers.^[2–6]^ The hallmark of HER2-positive breast cancer entails the amplification of the *HER2* gene and/or heightened expression of its corresponding kinase receptor proteins. These molecular alterations endow the cancer with a more aggressive nature, resulting in reduced disease-free survival and overall survival (OS).^[5,7–11]^

**Figure 1.**
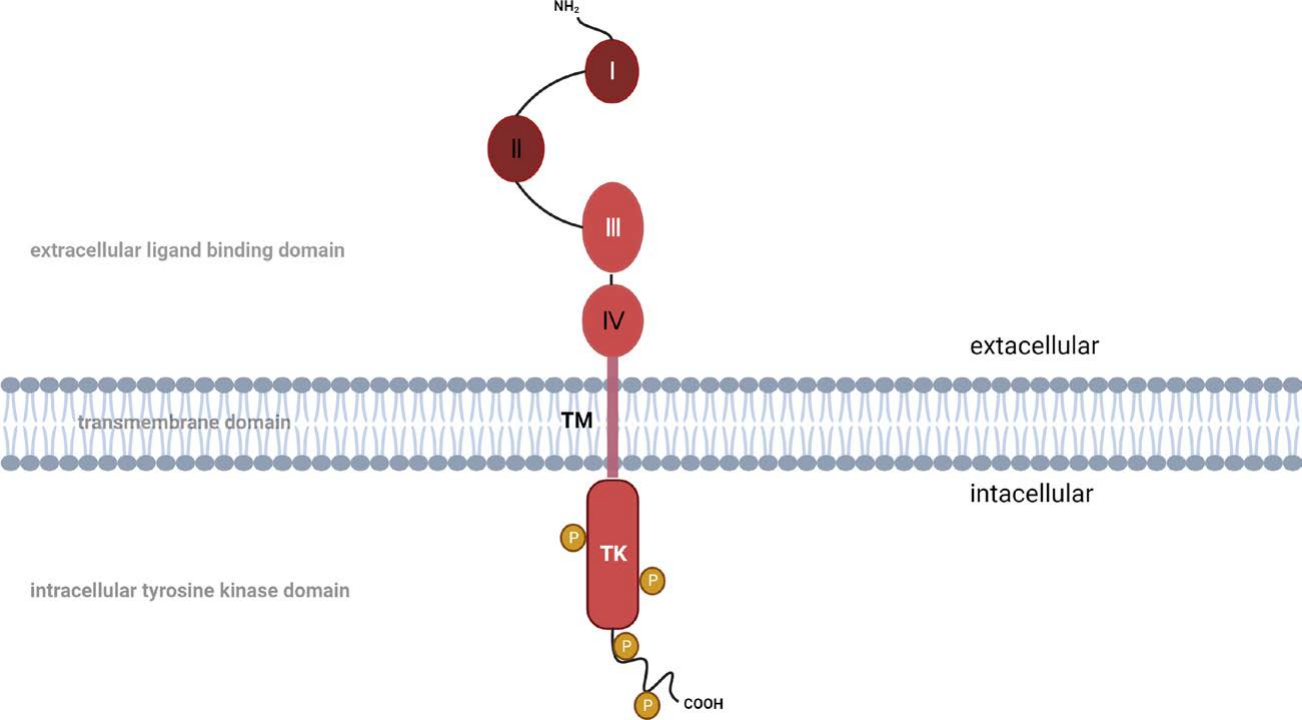
Structure of HER2 protein. The HER2 protein is formed by an extracellular ligand-binding domain (including structural domains I, II, III, and IV), a short transmembrane structural domain (TM), and an intracellular tyrosine kinase domain (containing the catalytic tyrosine kinase structural domain (TK) and the carboxy-terminal tail). Structural domains I and III of the extracellular ligand-binding domains are involved in ligand binding (except for the structural domain of HER2), and structural domains II and IV are two cysteine-rich regions. The carboxy-terminal tail of the intracellular tyrosine kinase domain has several tyrosine residues that can be phosphorylated. Created with BioRender.com. HER2/ErbB2 = human epidermal growth factor receptor 2.

The human epidermal growth factor receptor 2 (*HER2/ErbB2/neu*) oncogene, situated on chromosome 17 at band q21, is a part of the HER (HER1-4) family of growth factor receptors and encodes a transmembrane tyrosine kinase membrane receptor, HER2, 185,000 daltons in size,^[12]^ with a structure consisting of an extracellular domain, a membrane spaning domain and an intracellular tyrosine kinase^[7]^ (Fig. 1). HER2 lacks ligand-binding activity but binds to HER1, HER3, and HER4 activated by different ligands and is activated by homo- or heterodimerization at the cell membrane, thereby inducing a structural change in the receptor that initiates an intracellular phosphorylation cascade, whose main downstream targets include the mitogen-activated protein kinase (MAPK) and phosphatidylinositol-3-kinase (PI3K) signaling pathways.^[7,13]^ Phosphorylated (signaling) HER2 and dephosphorylated HER2 are in a dynamic equilibrium under normal conditions; this equilibrium regulates cell proliferation and survival and is engaged in the regulation of normal breast growth and development.^[14–16]^ When HER2 overexpression is aberrantly activated, it is often closely associated with cell invasion, migration, differentiation, angiogenesis, and chemoresistance and is a powerful biomarker for predicting the course of invasive clinical disease.^[17,18]^ For HER2-positive initial breast cancer, neoadjuvant therapy has become a common choice. Based on the subtype of the clinical tumor, primary treatments comprised endocrine therapy, anti-HER2 targeted therapy, and chemotherapy.^[19]^ By the end of 2006, landmark trial results demonstrated that chemotherapy coupled with curative HER2-targeting agent trastuzumab significantly benefits progression-free survival and overall OS in patients with early- and late- stages HER2-positive breast cancer.^[4]^

The serine/threonine-protein kinase AKT, commonly referred to as Protein Kinase B (PKB), is denoted as AKT and is divided into 3 isoforms: AKT1, AKT2, and AKT3, and composed of 3 conservative structural domains (the N-terminal PH structural domain, the central kinase structural domain, and a short C-terminal tail) and is a critical member in the PI3K signaling pathway.^[20,21]^ Activation of AKT involves conformational changes and phosphorylation. Its typical pathway for its activation is through stimulation of receptor tyrosine kinases (RTKs) or G protein-coupled receptors (GPCRs), causing activation of the class I PI3K family, which in consequence catalyzes the phosphorylation of phosphatidylinositol 4, 5-bisphosphate (PIP2) to generate phosphatidylinositol 3, 4, 5-triphosphate (PIP3). AKT is induced to be recruited by PIP3 accumulated on the membrane via its PH domain and then selectively activated by phosphoinositide-dependent kinase 1 (PDK1) and mammalian target of rapamycin complex 2 (mTORC2) at 2 regulatory sites (Thr308 and Ser473, respectively), changing its conformation and enhancing its activity. AKT, which is bound in its stable active conformation, is dispersed into the cytoplasm and nucleus and plays key roles in fundamental cellular activities including cell proliferation, metabolism, apoptosis, protein synthesis, and blood vessel formation.^[5,21,22]^

Here, we offer a comprehensive analysis of the importance of HER2 in breast cancer, specifically highlighting the influence of HER2 overexpression on the PI3K/AKT pathway in breast cancer. Furthermore, we provide a concise summary of the effects of the HER2-AKT pathway on the phenotype of HER2-positive breast cancers and the current status of targeting the HER2–AKT pathway for the medical treatment.

## 2. Significance of HER2 expression in breast cancer

In 1981, through transformation studies on DNA from ethylnitrosourea-induced rat glioblastomas, Shih C et al identified a new transforming gene, *neu*.^[23]^ The *neu* gene was later shown to be related to, but separate from, the *c-erbB* proto-oncogene.^[24,25]^ By screening the human genome with *v-erbB*, the avian erythroblastosis virus transforming gene encoding a 68,000 dalton truncated epidermal growth factor receptor (EGFR) and human EGFR (HER1) as probes, and a complementary DNA library, the human *erbB*-related gene *HER2* was isolated.^[26]^ The subsequent sequence analysis and chromosomal localization studies showed that *neu* and *HER2* share extensive homology and that *HER2* is the human homolog of *neu*, but both are distinct from EGFR.^[12,27–30]^

The biological relevance of HER2 as a RTK and a driver oncogene is indisputable. Studies have shown that HER2 amplification or aberrant activation is closely correlated with an aggressive phenotype in HER2-positive breast cancer, often orientated toward high recurrence rates and poorer survival outcomes.^[12,31,32]^ The research conducted by Tiwari et al uncovered that 88% (14 cases) of patients with *HER2*-amplified breast cancer had positive regional lymph nodes. Furthermore, among the 17 patients with HER2 amplification, 16 cases (94%) had developed metastatic disease at the point of diagnosis, These findings indicate a strong association between HER2 overexpression and early tumor metastasis in primary breast cancer.^[33]^ Meanwhile, in women with lymph node-negative breast cancer, irrespective of the level of HER2 overexpression, the likelihood of disease recurrence is magnified by a factor of 3.0 when compared to women with non-amplified *HER2* breast cancer, and the high overexpression group’s risk of breast cancer recurrence was 9.5 times higher than that in the normal expression group (*P* = .0001).^[34]^ Dysregulation of the RTK pathway, often due to receptor overexpression, gene amplification, or mutation, such as the overexpression of this receptor due to *HER2* gene amplification, is also a major factor that plays important roles in many cancers.^[27,35]^

The amplification/overexpression of HER2 has had a profound influence on the identification and subsequent advancement of specialized anti-HER2 therapies for individuals diagnosed with breast cancer. Back in 1998, the U.S. Food and Drug Administration (FDA) granted approval for the initial HER2-targeted medication, trastuzumab, in conjunction with paclitaxel, as a primary therapeutic option for HER2-positive metastatic breast cancer.^[36]^ The data indicated that adding trastuzumab to chemotherapy in women with HER2-positive metastatic breast cancer prolongs the time to disease progression (median, 7.4 vs 4.6 months; *P* < .001), increases objective response rate (50% vs 32%, *P* < .001), extends the duration of response (median, 9.1 vs 6.1 months; *P* < .001) and prolonged survival (median survival, 25.1 months vs 20.3 months; *P* = .046).^[37]^

## 3. HER2 signaling pathway

**Figure 2.**
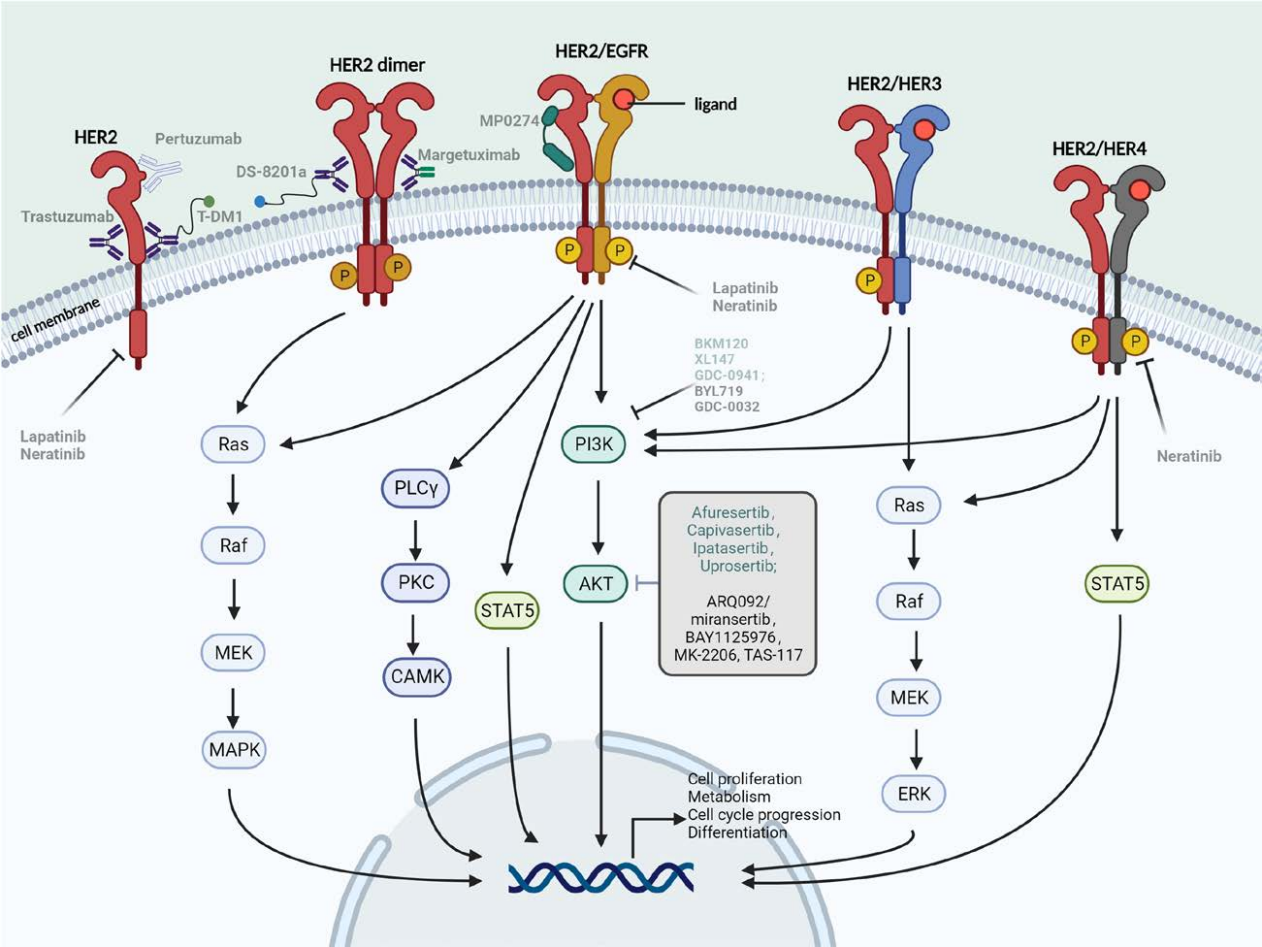
The HER2 network and drugs targeting the HER2–AKT signaling pathway for the therapy of HER2-positive breast cancer. The HER signaling system mainly consists of HER1–4 receptors and 11 ligands. HER2, although lacking ligand, exists in an open state in the membrane, HER1, HER3, and HER4 bind to ligands and are induced to undergo conformational changes that promote heterodimerization with HER2 or the formation of homodimers with itself to activate tyrosine kinases and initiate phosphorylation cascades. The downstream signaling pathways triggered by the HER receptor family mainly include the PI3K/AKT, MAPK, and PLCγ pathways, and the activated downstream kinase modifies and phosphorylates transcription factors, transcripts, and elements of the cell-cycle machinery, enabling them to modify the expression of genes and contribute to the proliferation and survival of tumor cells, as well as metastatic and angiogenic capacity. Created with BioRender.com. HER2/ErbB2 = human epidermal growth factor receptor 2, HER3/ErbB3 = Human epidermal growth factor receptor 3, HER4/ErbB4 = Human epidermal growth factor receptor 4, MAPK = mitogen-activated protein kinase, PLCγ = phospholipase Cγ.

The EGFR tyrosine kinases family comprises 4 receptor tyrosine kinases (HER1/EGFR/ErbB1, HER2/ErbB2, HER3/ErbB3, and HER4/ErbB4), which synergistically initiate numerous cellular signaling pathways.^[8,38]^Several interconnected and overlapping modules make up the HER signaling network.^[39]^ HER1, HER3, and HER4 are activated at the cell membrane through binding with distinct ligands, inducing conformational changes in the receptor to promote heterodimerization with HER2 or the formation of a homodimer with itself to activate tyrosine kinases (TKs)^[7,8]^ (Fig. 2).

Despite lacking any known ligand binding capability, HER2 exhibits the most potent catalytic kinase activity within the HER family (i.e., a proliferative response triggered by binding to related ligands: HER2 > HER1 > HER4 > HER3).^[7,32]^ HER2 is the preferred dimerization chaperone for other members of the HER family or for itself and is activated by either homodimerization with other HER2 receptors in a non-ligand dependent manner or by ligand-mediated heterodimerization with other members of the HER family through ligand binding. The EGFR/HER2 and HER2/HER3 combinations are the most significant pairs within heterodimers that include HER2, and have a profound impact on cell function and disease.^[40]^ Only in the absence of HER2 neu differentiation factor-activated HER3 or HER4 dimerize heterodimerically with HER1, and epidermal growth factor (EGF)- and β-cytokinin-inspired HER3 activation is impaired in the deficiency of HER2.^[41]^ Although HER2 and HER3 are incomplete signaling molecules, with no known ligand for HER2 and a lack of kinase activity for HER3, extensive coimmunoprecipitation of affinity-tagged HER3 with HER2 and the relative hyperphosphorylation of HER2 upon neu differentiation factor binding to HER3 demonstrated that of the 3 other HER proteins with which HER2 interacts, HER3 is the most efficient member and the major binding partner of HER2 (reverse activation of HER2 by HER4 is the least efficient, whereas transactivation of HER3 is the most efficient interaction). In contrast, transphosphorylation between HER3 and HER1 is relatively limited. Furthermore, of the 4 HER proteins, only HER2 exhibited constitutive tyrosine phosphorylation upon overexpression.^[32]^ Thus, HER2 overexpression in *HER2*-amplified breast cancer leads to the formation of multiple HER2 heterodimers and HER2 homodimers (HER2 is more biased toward the formation of heterodimers), resulting in stronger cellular signaling, enhancing the response to growth factors and malignant growth.^[7,32,42]^

The HER2 downstream signaling pathways include the PI3K/AKT, MAPK and phospholipase Cγ (PLCγ) pathways. The PI3K/AKT pathway mediates cell survival, and the Ras/ERK1/2 and PLCγ pathways are engaged in cell proliferation.^[38,43]^ The HER family also controls tumor cell proliferation and apoptosis through the signal transducer and activator of transcription 5a/5b(STAT5A/B), p38 and c-Jun amino-terminal kinase (JNK) pathways, they modulate caspase activation and PARP-1 cleavage via Bcl2.^[44,45]^ In a considerable proportion of breast cancers, an increase in MAPK expression and activation of the Ras/Raf/MEK/ERK1/2 MAPK pathway can be observed, with its activity levels being 5 to 10 times higher compared to benign conditions like fibroadenomas and fibrocystic diseases.^[46–48]^ Furthermore, the activation of the MAPK pathway is associated with poor response to hormone therapy and decreased survival rates in clinical breast cancer patients.^[49–51]^ There exists a strong negative correlation between the PI3K/AKT and Ras/ERK pathways, whereby inhibition of 1 pathway generally triggers activation of the other pathway.^[52,53]^ Research indicates that PI3K can be activated as a downstream effector of Ras.^[54]^ Activated ERK suppresses RTK-mediated PI3K/AKT signaling induction through phosphorylation of the scaffold adapters GAB1 and GAB2.^[21]^ Simultaneously, the Ras-Raf-MEK-ERK and PI3K-AKT pathways cross-talk at the levels of Raf-1 and AKT, where high doses of insulin-like growth factor-I (IGF-I) rapidly activate AKT, subsequently inhibiting Raf activation via phosphorylation at Ser-259.^[55]^ Moreover, mTORC1 inhibition in human cancers triggers Ras/MAPK pathway activation via the S6K-PI3K-Ras feedback loop, thereby promoting AKT activation and ERK phosphorylation.^[56]^ Hence, the reciprocal inhibition and feedback regulation between the PI3K/AKT and MAPK pathways not only participate in crucial processes such as normal cell growth and proliferation, but also increase the challenge of comprehensive blockade of the HER2/PI3K/AKT and MAPK pathways with a singular medication.

In addition, other separate mechanisms that can actuate the HER pathway add to the complication of the HER signaling system. First, additional membrane receptors, like insulin-like growth factor-I receptor (IGF-IR)^[57]^ and MET,^[58]^ may heterodimerize with HER2 and initiate a phosphorylation cascade reaction. Prolactin can activate the downstream signaling pathways of EGFR and HER2 through crosstalk within the prolactin receptor (PRLR)^[59]^ and EGFR/HER2. In breast cancer cells simultaneously expressing HER2 and ER, there exists an inverse association between ER expression and HER pathway activation,^[60,61]^ and overactivation of protein kinases in signaling pathways downstream of the HER receptor (e.g., MAPK and PI3K/AKT) leads to a significant reduction or total loss of ER expression and activation.^[62–65]^ Anti-HER2 treatment leads to enhancement in ER expression and activity.^[61,66]^ ER can also initiate HER signaling when combined with estrogen,^[67]^ and the bidirectional crosstalk that exists between the 2 receptors may act as an escape route from HER2 inhibition.^[61]^

Alterations in signaling molecules in the HER receptor family of signaling pathways, e.g., deletion of the tumor suppressor genes inositol polyphosphate 4-phosphatase type II (*INPP4B*) and phosphatase and tensin homologue (*PTEN*), are also known to actuate the PI3K pathway.^[68]^ These alternative or cross-talk mechanisms that activate the HER signaling pathway network could partially explain the resistance to treatment targeting only HER2. The HER signaling pathway network includes the HER1, HER2, HER3, HER4 receptors and the 4 complete transmembrane isoforms of HER4 (JMaCTa, JMaCTb, JMbCTa, and JMbCTb), 11 ligands (EPG, EGF, AR, TGF, HB-EGF, EPR, β-cytokinin, Nrg-1, Nrg-2, Nrg-3, Nrg-4) and 28 homo/heterodimers, with 614 possible receptor combinations,^[38]^ in spite of this, there are multiple feedback loops and multiple levels of control in the MAPK and PI3K pathways. The HER signaling pathway system is thus complicated, redundant, able to be fine-tuning, adaptive, and tough to block entirely^[8]^ (Fig. 2).

## 4. PI3K/AKT signaling pathway

PI3K, which consists of 8 isoforms in mammals, is a plasma membrane-bound enzyme, the categorization of these enzymes into 3 classes (I-III) is based on their structural features and substrate specificity: Class I PI3K is a heterodimeric enzyme consisting of a regulatory subunit (p85α, p85β, p85γ) and an activated catalytic subunit (p110α, p110β, p110δ, p110γ) and forms heterodimeric enzymes, including Class IA (PI3Kα, PI3Kβ, and PI3Kδ) and Class IB (PI3Kγ),^[5,35]^ Class I PI3Ks are the most characteristic cancer-related proteins and are activated primarily by RTK signaling, while some p110β-containing enzymes can also be initiated by GPCR,^[69,70]^ and the activated Class I PI3Ks primarily generate PIP3 from PIP2; Class II PI3Ks are high-molecular-mass monomers comprising 3 different enzymes types (PI3KC2α, PI3KC2β, and PI3KC2γ), in vitro, Class II PI3Ks exhibit a preference for producing phosphatidylinositol-3-phosphate (PI-3-P) and phosphatidylinositol-3,4-biphosphate (PI-3,4-P2). Additionally, in vivo, they are capable of generating PI-3-P, PI-3,4-P2, and potentially PIP3; and Class III PI3Ks have only 1 member, VPS34 (aka PI3K-C3), the only PI3K expressed in all eukaryotes, which generate PI-3-P from phosphatidylinositol (PI) in vivo.^[35,71]^

**Figure 3.**
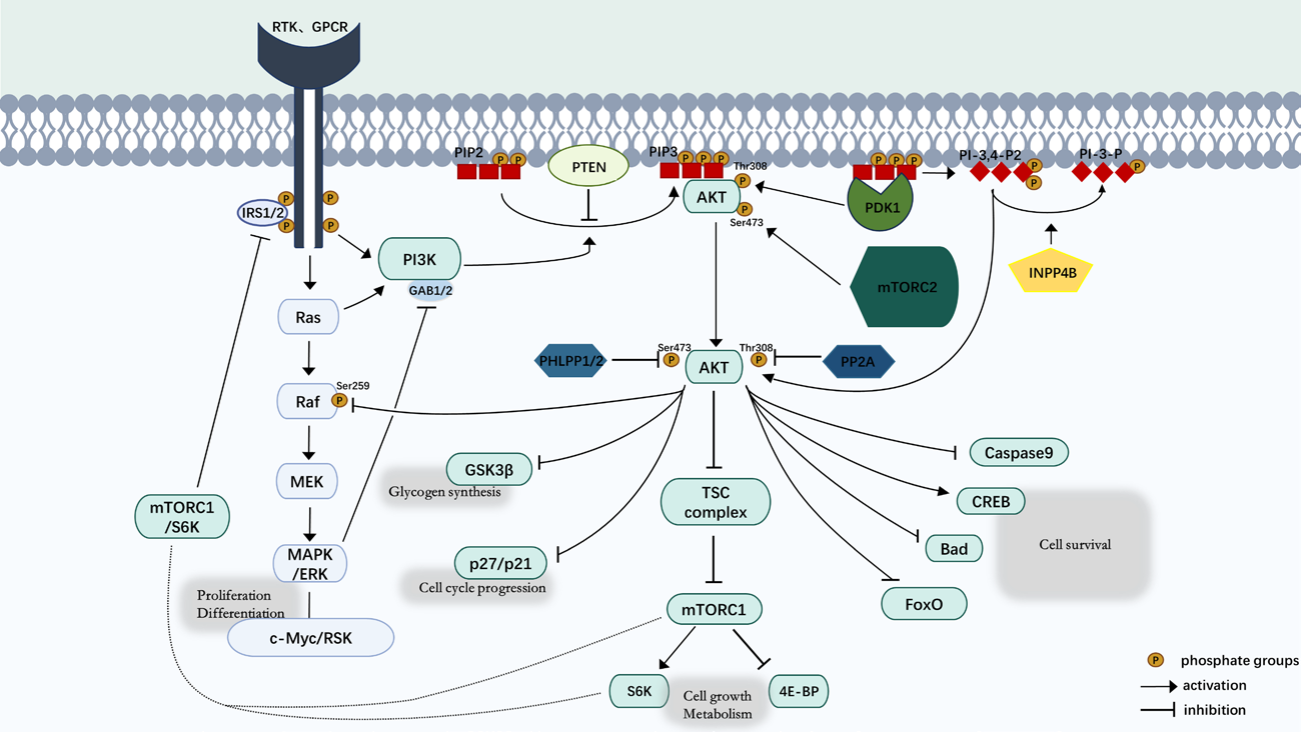
The classic signaling pathways PI3K/AKT/mTOR and Ras/Raf/MEK/ERK MAPK, along with their downstream signaling molecules and mediated cellular processes. In quiescent cells, PTEN maintains low levels of PIP3, leading to AKT inactivation. Activated RTK (ligands: insulin, growth factors, hormones) and GPCR (chemokines) recruit and activate PI3K, which catalyzes the conversion of PIP2 to PIP3. PIP3 recruits AKT and PDK1 to the membrane via their PH domains, followed by phosphorylation of AKT at T308 and S473 by PDK1 and mTORC2, respectively. Activated AKT activates or inhibits downstream signaling molecules (GSKβ, mTORC1, p27, p21, Bad, FoxO, etc.) through phosphorylation to participate in cell proliferation and metabolism. PTEN and INPP4B negatively regulate AKT activity by dephosphorylating PIP3 and PI-3,4-P2. Additionally, PP2A and PHLPP1/2 dephosphorylate AKT at T308 and S473, respectively. mTORC1 participates in negative feedback regulation of RTK-PI3K signaling by inducing IRS1/2 degradation, leading to dephosphorylation of AKT and ERK. In the Ras/Raf/MEK/ERK pathway, inactive Ras-GDP binds to the membrane in quiescent cells. Upon activation of RTK (growth factors) or GPCR (polypeptide hormone, neurotransmitter, chemokine), Ras-GDP is recruited to the membrane and converted to Ras-GTP (active state), which then recruits and activates Raf, initiating the MAPK cascade to regulate cell proliferation and differentiation. Activated ERK inhibits the PI3K/AKT signal via GAB1/2. There’s also cross-talk and convergence between the PI3K/AKT and Ras/Raf/MEK/ERK pathways, with Raf-AKT interactions regulating downstream processes. AKT = protein kinase B, ERK1/2 = extracellular signal-regulated kinase 1/2, GPCRs = G protein-coupled receptors, GPCRs = G protein-coupled receptors, IRS = insulin receptor substrate, MEK = extracellular signal-regulated kinase (ERK) kinase, MAPK = mitogen-activated protein kinase, PLCγ = phospholipase Cγ, PI-3,-P2 = Phosphatidylinositol-3,4-biphosphate.

AKT serves as the major downstream effector of the PI3K pathway, activated PI3K by extracellular stimulation leads to the recruitment and activation of AKT in almost all cells and tissues; therefore, PI3K and its lipid derivatives are often regarded as necessary for the proper activation of AKT.^[21]^ Upon activation of class I PI3Ks, they phosphorylate PIP2 to produce the second messenger PIP3, a progression that can be turned around by PTEN. PIP3 on the cell membrane forms a docking site for the PH structural domains of PDK1 and AKT, inducing their recruitment. When bound to PIP3 on the plasma membrane, PDK1 directly activates and phosphorylates the T308 site of AKT. Additionally, mTORC2, which is constitutively active on the plasma membrane, phosphorylates the S473 site of AKT. Both PDK1 and mTORC2 are essential for fully activating AKT, nevertheless, their phosphorylation of AKT operates independently, and the phosphorylation status impacts the specificity of AKT substrates.^[22,70]^ Activated AKT diffuses into both the cytoplasm and the nucleus, phosphorylating various effectors with distinct functions. The first identified direct substrate of AKT is glycogen synthase kinase 3 beta, whose phosphorylation weakens its inhibition of cyclin D1, thereby enhancing proliferation. The PI3K/AKT signaling pathway triggers mammalian target of rapamycin complex C1 (mTORC1) activation, a crucial downstream effector, through phosphorylation-induced inhibition of tuberous sclerosis complex 2 (TSC2), TSC2 facilitates the transformation of Ras-related GTPase Rheb from its active Rheb-GTP state to the inactive Rheb-GDP form, active Rheb-GTP effectively activates mTORC1, thereby positioning the TSC complex, which includes TSC2, as an inhibitor of mTORC1. activated mTORC1 acts to suppress S6K and eukaryotic translation initiation factor 4E-binding protein, promoting ribosome and protein synthesis involved in the synthesis metabolism of proteins, lipids, nucleotides, and more. Additionally, AKT phosphorylation leads to the inactivation of p21 and p27, promoting cell proliferation, and negatively regulates apoptosis by blocking the activity of pro-apoptotic factors Bax, Bad, and Forkhead box protein O transcription factors^[21,22]^(Fig. 3).

Under normal circumstances, the activity of AKT is also regulated by lipid phosphatases and protein phosphatases. Lipid phosphatases INPP4B hydrolyzes PI-3,4-P2 to generate PI-3-P, restricting AKT membrane recruitment, exerting a PTEN-like effect.^[72]^ PH domain and leucine-rich repeat protein phosphatases 1/2 (PHLPP1/2) and protein phosphatase 2A (PP2A) dephosphorylate S473 and T308 residues, directly inhibiting AKT activity.^[73,74]^ Moreover, the PI3K/AKT signaling pathway is suppressed by negative feedback regulation. One such pathway is the mTOR–raptor-dependent pathway, which interferes with insulin receptor substrate (IRS)-mediated PI3K activation, mTORC1 activation induces the degradation of IRS1/2 by phosphorylating mTORC1 or S6K1, consequently suppressing PI3K activation.^[75]^ Under the regulation of these events, the PI3K/AKT/mTOR signaling pathway is in equilibrium.

The dysregulation of the PI3K/AKT signaling pathway occurs frequently in human cancers, often attributed to RTKs and somatic mutations in specific components of this signaling pathway.^[76]^ Approximately 30% to 40% of cases exhibit mutations in *PIK3CA* in breast cancers, which induces overactivation of the α isoform of PI110K (p3α),^[5]^ and aberrant stimulation of the PI3K/AKT pathway has been linked in tumor growth, angiogenesis, and survival^[35]^ and is also implicated in resistance to EGFR-mediated endocrine therapy and other forms of directed therapy, such as targeting HER2 resistance.^[77,78]^

## 5. Common genetic alterations in HER2-positive breast cancer

The activation and oncogenic potential of HER2 have been well established in breast cancer, wherein the main mechanism of HER2 activation is *HER2* gene amplification leading to an excessive expression of HER2 at the cell membrane. For HER2, gene overexpression (copy number gain) is as yet the most prevalent genomic alteration and is usually accompanied by protein overexpression.^[79]^ In recent years, there have been discoveries of additional changes in the *HER2* gene that result in the activation of proteins, with *HER2* mutations being the most significant variant. Typically, *HER2* mutations exhibit activating characteristics and are frequently observed in conjunction with *HER2* gene amplification.

In the 2018 updated ASCO/CAP guidelines, an ERBB2-positive breast cancer is defined by a ratio of 2 to chromosome enumeration probe 17 (CEP17) in fluorescence in situ hybridization, a copy number of 6 in ERBB2, or a score of 3 + for HER2 immunohistochemistry.^[80]^ The Cancer Genome Atlas project has shown that there is not the same genomic profile for all HER2-positive breast cancers. Intrinsic subtyping of breast cancer using the PAM50 gene expression assay allows tumors to be divided into luminal A, luminal B, *HER2*- amplified (approximately 10–15%), basal-like and normal.^[81]^ The *HER2*- amplified subtypes are frequently characterized by HER2 amplification (80%), in addition to significantly higher expression levels of many RTKs, including FGFR4, HER1 and genes included in the *HER2* amplicon (e.g., *GRB7*), and have high levels *TP53* (72%) and *PIK3CA* (39%) mutations, as with mutations in additional genes (e.g., *PTEN, PIK3R1*) (with markedly lower frequencies, commonly < 10%); the luminal subtype expresses high levels of characteristic luminal genes including *ESR1, GATA3* and *BCL2*.^[68]^

*HER2* mutations (~3%) are rare compared with amplifications, but they can induce oncogenic effects by constitutively activating HER2 or by promoting the dimerization of HER2 with other constituents of the HER family. Approximately 70% of HER2 mutations happen in the structural domain of the kinase at amino acids 755 and 781 (between exons 19 and 20), and an additional 20% occur at amino acids 309 or 310 (exon 8) in the extracellular domain.^[28,36]^ The 3 mutations with the highest frequencies in *HER2*-amplified breast cancer are known as V777L, L755S, and D769Y, all of which are predicted to be driver mutations that appear to be induced (at least in part) by HER2-targeted and endocrine therapies and are prevalent in metastatic tumors.^[10,12,82]^ In addition, p95HER2^[50]^ (a truncated receptor does not possess the extracellular epitope identified by anti-HER antibodies) and Δ16HER2 (a splicing isoform heterodimer encoding a deletion of an extracellular structural domain encoded in exon 16, leading to HER2 homodimerization and constitutive stabilization of HER2 signaling) are also present in patients with HER2-positive breast cancer.^[42]^ Presentation of p95HER2 has been measured in approximately 30% of breast cancers, and among HER2-positive breast cancer patients, those expressing p95HER2 have a poorer prognosis than those expressing full-length HER2.^[83,84]^ The marked increases in Δ16HER2 in *HER2*-amplified cancers have been thought to be mechanisms of HER2 tumorigenesis, accounting for 4% to 9% of all HER2 transcripts.^[7]^ According to data displayed on cBioPortal, approximately 2% of breast cancer patients carry a HER3 gene mutation.^[85,86]^ However, HER2 and HER3 mutations are not frequently co-expressed in HER2 amplification breast cancer cells.^[87]^

Somatic mutation analysis from The Cancer Genome Atlas showed that except for *HER2* amplification, primary *HER2*- amplified breast cancers have a wide variety of rare mutations (<10%), with *HER2* (3.6%) included; nevertheless, *PIK3CA* (~31%), and *TP53* (40%) mutations were the most common.^[10]^ Seventy-three percent of the mutations in *PIK3CA* were H1047R (35%), E545K (17%), E542K (11%), N345K (6%), and H1047L (4%).^[88]^ *PIK3CA* gene mutations are the most widespread genetic alterations other than loss of *PTEN* and are related to increased activity of the PI3K/AKT signaling pathway, which enhances cancer cell proliferation and invasion.^[89,90]^

*AKT* mutations are less commonly reported in breast cancer, with less common *AKT* substitutions (2–4%) or amplifications (5–10%) present.^[91]^ The PH domain of AKT1 is where the predominant *AKT* mutation in HER2-positive breast cancer commonly takes place. This mutation involves the replacement of glutamic acid at position 17 (E17K) with a lysine residue, leading to an increase in kinase activity. Meanwhile AKT3 was found to be the most commonly amplified AKT isoform in breast cancers and has been investigated predominantly in treatment-resistant triple-negative subtypes, though its role in *ERBB2*- amplified breast cancer is still only partially understood.^[22]^ In a 2008 mutational study using a mass spectrometry-based single-nucleotide polymorphism approach to identify the *AKT1 E17K* mutation and *AKT2* and *AKT3* equivalents, the presence of *AKT1 E17K* mutation was identified in only 6 out of 418 breast cancers (1.4%) and was limited to hormone receptor (HR)^+^ breast cancers with ER and PR expression (6 out of 186 cases, 3.2%). No *AKT1 E17K* mutation was found in 75 HER2 + or 111 HR-/HER2-breast cancers. *AKT2 E17K* and *AKT3 E17K* mutations were not found in any of the samples. Moreover, it is worth mentioning that the occurrence of *AKT1 E17K, PIK3CA*, and *PTEN* mutations did not coincide within the same cases, they were mutually exclusive.^[90]^

## 6. HER2 overexpression and PI3K/AKT pathway alterations

In previous studies, it has often been found that HER2 overexpression aberrantly activate the PI3K/AKT signaling pathway in HER2-positive breast cancer. We have summarized several pathways that result in the aberrant activation of AKT by HER2, including HER2 amplification and/or mutation, *PIK3CA* mutation, other RTK (HER3) alterations, or alterations of other components of the AKT pathway.

### 6.1. HER2 overexpression or mutation

In breast cancer, overactivation of the PI3K/AKT cascade may occur when RTKs (such as HER2) undergo amplification or acquire functional mutations.^[22,91,92]^ When HER2 is overexpressed or amplified, it triggers tumorigenesis by the spontaneous generation of homodimers or heterodimers with additional HER family members, and HER2 phosphorylation enables activation of the PI3K/AKT pathway, with evidence supporting that HER2, when overexpressed, can have an activating effect on AKT and induce cell transformation independent of its heterodimeric chaperones (including HER3),^[93]^ The direct binding of HER2-HER3 heterodimers with the p85 subunit of PI3K mediates PI3K signaling. However, in response to the overexpression of HER2 genes, there is a spontaneous increase in the formation of these heterodimers, and plays a significant role in the abnormal activation of the downstream PI3K/AKT signaling pathway.^[5,79,94,95]^ In addition, a splice heterodimer of HER2, ΔHER2, has enhanced ligand-dependent signaling activity and enhanced transforming potency due to the lack of cysteine residues, leading to conformational changes in the extracellular structural domain of the HER2 receptor, which generates homodimers capable of causing cell transformation, leading to the stabilization of constitutive HER2 signaling.^[96]^ p95HER2 has the potential to enhance signaling transduction activity; though its expression and effect in human breast cancer have not been thoroughly explored, it has an essential role as a biomarker of metastasis and poor outcome in breast cancer.^[83,97]^

### 6.2. *PIK3CA* mutation

In a comprehensive meta-analysis of cancer genome sequencing studies, *PIK3CA* and *PTEN* were reported to be the second and third most frequently mutated genes in human cancers,^[98]^ with a *PIK3CA* mutation rate of 31% in HER2-positive breast cancer.^[88]^ Around 50% of HR + breast cancer cases and approximately 25% of triple-negative breast cancer cases display an excessively active PI3K/AKT pathway, which is primarily maintained by *PIK3CA* point mutations in HR + tumors and *PTEN* deficiency in triple-negative breast cancer, whereas deregulation of the PI3K/AKT signaling pathway in HER2-positive breast cancers is mainly due to *PIK3CA* mutations.^[91,94]^ *PIK3CA* mutations can also additionally enhance PI3K pathway activation supported by other oncogenes (e.g., HER2).^[77]^

### 6.3. HER3

The experimental data from Lee-Hoeflich et al indicates a correlation between overexpression of HER2 and activation of HER3 in human breast cancer, and in HER2-positive breast cancer tissue, HER3(Tyr1289) is observed to be preferentially phosphorylated,^[99]^ and approximately 50% to 70% of breast cancer patients display elevated levels of the HER3.^[100]^ HER2 exhibits deficiencies in the binding site of the p85 subunit in PI3K, while HER3 possesses 7 motifs that contain tyrosine phosphorylation sites capable of binding to p85, consequently, HER3 is able to directly initiate the activation of PI3K/AKT pathway.^[101,102]^ Research has shown that for HER2-positive breast cancer, HER2 often aberrantly activates AKT through HER3 and PI3K, and the transactivation of HER3 and PI3K/AKT appears to make HER2 overexpression cancer-causing potential.^[7,103]^ Furthermore, the *HER3* mutation at the E928G site leads to elevated levels of p-AKT in cells.^[87]^ This may be due to the presence of PI3K regulatory subunit sites within the kinase region where the E928 residue is located. When this residue changes, more PI3K binding subunit sites are recruited, resulting in an increase in activity of the PI3K/AKT signaling pathway.^[104,105]^

### 6.4. Other changes (e.g., PTEN)

In HER2- positive breast cancer, the frequencies of mutations (e.g., deletions, substitutions, amplifications) in the *AKT* or *PTEN* genes are low (usually < 10%), and AKT is also directly activated in response to mutational deletions of *PTEN* and *INPP4B* lipid phosphatase and copy number alterations of *AKT* isoforms.^[22]^ Lu et al have validated that the lack of PTEN function in breast cancer cells induces an increase in the PI3K signaling cascade, with increased levels of basal phosphorylation of multiple components, as well as prolonged signaling in response to ligands through the PI3K cascade, these alterations can be reversed by reintroducing functional PTEN.^[106]^ In addition, approximately 10% of breast cancers show amplification of the *RPS6KB1* gene, responsible for encoding the P70S6 protein kinase. This gene is closely linked with HER2 overexpression, possibly because they are both located on chromosome 17’s short arm (17q), which ultimately causes aberrant activation of the PI3K signaling pathway.^[107,108]^

## 7. Role of the HER2–AKT pathway in breast cancer

HER2 amplification has been shown to positively enhance PI3K/AKT/mTOR signaling, which is in charge of regulating various aspects of tumor biology, for instance tumor cell proliferation, invasion, and differentiation.^[109]^ However, the specific function of the HER2–AKT pathway in breast cancer remains to be elucidated. In the study conducted by Kim et al., Disulfiram/copper drug hinders the HER2-AKT pathway in breast cancer cells resulting in decreased levels of HER2, p-HER2 (Tyr1221), and p-AKT (Ser473), this process triggers apoptosis and eliminates cancer stem cell-like properties.^[110]^ In a similar vein, when the HER2 gene was knocked down in HER2 + breast cancer cell lines using RNA interference (RNAi), cell proliferation was also inhibited by up to 3- to 5-fold.^[99]^ These findings are in accordance with previous studies showing that transduction of HER2-positive breast cancer cells with an adenovirus encoding active (myristoylated) Akt (Myr-Akt) avoided HER2 antibody herceptin-induced inhibition of cell proliferation in BT474 and apoptosis in SKBR3.^[111]^ Similarly, ansamycin degraded HER2 and inhibited tumor growth by inducing apoptosis and growth arrest in HER2-positive cells via the HER3/PI3K/AKT-dependent pathway,^[112,113]^ which could likewise illustrate the promotional function of the HER2–AKT pathway in cell proliferation.

HER2-mediated modulation of PI3K/AKT signaling pathway activity not only plays a key role in promoting cell proliferation and inhibiting cell death, but has also been shown to mediate multidrug resistance in human breast cancer. It has been found that the HER2-mediated increases in resistance to multiple chemotherapeutic agents were associated with increased AKT kinase activity and that chemoresistant cells with increased AKT activity were sensitive to selective inhibition of PI3K using specific inhibitors or dominant-negative expression vectors, suggesting that its effects are mediated via PI3K; thus, appropriate combinations of traditional chemotherapeutic agents with new-generation signal transduction inhibitors of the HER/PI3K/AKT pathway may provide clinical advantages in treating breast cancer patients.^[114]^

## 8. Drugs targeting the HER2–AKT pathway

### 8.1. HER2-targeted drugs

Several types of HER2-targeted drugs have been created, such as monoclonal antibodies (trastuzumab and pertuzumab), small-molecule tyrosine kinase inhibitors (lapatinib, neratinib, and afatinib), and antibody–drug conjugates (trastuzumab emtansine T-DM1 and trastuzumab deruxtecan DS-8201a).^[115–117]^ The humanized monoclonal antibody trastuzumab blocks breast cancer cell proliferation by targeting subdomain IV in the extracellular structural domain of HER2 to prevent HER2 homoheterodimerization and heterodimerization, thereby inhibiting the downstream signaling activation of HER2 and inducing antibody-dependent cytotoxicity,^[59,118,119]^ effects that are of landmark significance in improving the prognosis of patients with both early- and late -stage HER2-positive breast cancer.^[42,120,121]^ Pertuzumab specifically targets a site on the extracellular domain II of the HER2 receptor, blocking the interaction between HER2 and other HER receptors, notably HER3, this prevents the formation of HER2 dimers, effectively inhibiting their activity.^[122]^ The combination of Pertuzumab and Trastuzumab provides a 50% clinical advantage in treating patients with metastatic HER2-positive breast cancer, without raising the risk of cardiac toxicity.^[123]^ Small-molecule tyrosine kinase inhibitors bind to the ATP-binding site of the HER receptor to directly inhibit its kinase activity, blocking signaling in the Ras/Raf/MAPK and PI3K/AKT pathways, resulting in increased apoptosis and reduced cell proliferation. Lapatinib stands as the leading HER2 TKI in clinical practice, in a Phase III trial, its combination showcased superior progression-free time when compared to the sole utilization of capecitabine (8.4 months vs. 4.4 months; *P* < .001).^[122,124]^ T-DM1 is currently the only approved antibody–drug conjugate for advanced HER2-positive breast cancer,^[125]^ consisting of trastuzumab linked to a potent anti-microtubule agent DM1 through a nonreducible thioether linkage (SMCC).^[126]^ T-DM1 interacts with the HER2 receptor, causing internalization and subsequent release of DM1 inside the cell. This focused strategy ensures accurate delivery of powerful chemotherapy specifically to HER2-positive cells.^[122,127]^ T-DM1 has shown exciting results in trastuzumab-treated patients with advanced HER2-positive breast cancer, demonstrating promising antitumor activity with response rates approaching 50% and mild and reversible toxic effects.^[128]^ DS-8201a is composed of the trastuzumab linked with a topoisomerase I inhibitor (an exatecan derivative) using a tetrapeptide-based, cleavable linker^[4,129,130]^ that can kill not only targeted cells but also surrounding cells (bystander effect).^[131]^

In addition, Numerous novel drugs that specifically target HER2 are presently under investigation in clinical trials. These include Fc-optimized monoclonal antibodies like margetuximab, tyrosine kinase inhibitors such as tucatinib and pyrotinib, and biosimilars of pertuzumab such as trastuzumab-dkst, trastuzumab-dttb, trastuzumab-pkrb (CT-P6), trastuzumab-qyyp, and trastuzumab-anns, and a proprietary anchoring protein repeat protein (DARPin)-based drug, MP0274, that targets HER2.^[28,42,79]^ HER2-targeting antibodies counteract HER2-positive cancer cells through a variety of mechanisms, including activating antibody-dependent cell-mediated cytotoxicity, which relies on the interaction of the antibody’s Fc domain with the Fcγ receptor expressed by immune effector cells.^[132]^ Margetuximab is a second-generation anti-HER2 immunoglobulin G1 (IgG1) monoclonal antibody that targets the same epitope as trastuzumab and possesses a modified Fcγ region, thereby retaining the direct anti-proliferative activity of trastuzumab and similar Fc-independent anti-proliferative effects.^[132,133]^ Unlike T-DM1, which has shown notable benefits exclusively among patients with advanced HER2-high expressing breast cancer, Margetuximab presents a unique practicality for individuals with low levels of HER2 expression or those who possess the low-affinity CD16A allele.^[133]^ MP0274 exhibits 2 domains capable of binding to separate locations on HER2, this unique binding mode induces a distinct pro-apoptotic impact on HER2. Notably, the wild-type PI3K signaling pathway plays a vital role in the efficacy of MP0274.^[134]^ Trastuzumab biosimilars are biologic products sourced from living cells, showcasing comparable pharmacokinetic and pharmacological characteristics to the original reference medication. Notably, trastuzumab-dkst achieved the distinction of being the initial FDA-approved trastuzumab biosimilar.^[28,135]^ In HER2-positive breast cancer metastases treatment, paclitaxel combined with trastuzumab and pertuzumab is typically the initial therapy. Following that, T-DM1 is commonly used as a second-line therapy based on findings from the EMILIA trial (ClinicalTrials.gov identifier NCT00829166). However, the selection of therapy becomes uncertain for third-line and subsequent treatments^[4,136,137]^ (Table 1).

**Table 1:** HER2-targeted drugs.

Agent	Target	Mechanism of action	Highest R&D status (global)
Trastuzumab	Extracellular near-membrane subdomain IV of HER2	Prevents HER2 homodimerization and heterodimerization by binding to subdomain IV of HER2	Clinically approved for HER2-positive breast cancer metastasis patients who have undergone one or more chemotherapy regimens, or in combination with paclitaxel for treating HER2-positive breast cancer metastasis patients without prior chemotherapy. (1998-09-25)
Pertuzumab	Extracellular near-membrane subdomain II of HER2	Prevention of HER2 homodimerization and heterodimerization by combining with subdomain II of HER2	Clinically approved for the treatment of HER2-positive metastatic breast cancer patients who are treatment-naive, in combination with trastuzumab and docetaxel. (2012-06-08)
Trastuzumab emtansine (T-DM1)	Extracellular near-membrane subdomain IV of HER2 + Tubulin	T-DM1 is bound to the extracellular subdomain IV of HER2 and internalized into the cytosol, followed by phagocytosis by intracellular lysosomes and release of DM1. DM1, an efficient antimitotic drug, exhibits a comparable microtubule binding mechanism to colchicine alkaloids.	Clinically approved for HER2-positive metastatic breast cancer patients with prior trastuzumab and taxane therapy, separately or in combination. (2013-02-22)
Trastuzumab deruxtecan (DS-8201a)	Extracellular near-membrane subdomain IV of HER2 + TOP I	DS-8201a binds to subdomain IV of HER2 and is internalized into the cell, where it is cleaved by lysosomal enzymes such as histone B and L. Topoisomerase I inhibitors cause double-stranded DNA breaks and initiate apoptosis by binding to the topoisomerase I-DNA complex, forming a cleavable complex^[125]^	Clinically approved for adult patients with previously treated with two or more anti-HER2 therapies but still have inoperable or metastatic HER2-positive breast cancer. (2019-12-20)
Lapatinib	HER1, HER2	Inhibition of receptor signaling activation processes by binding to the ATP-binding pouch of the HER1/HER2 protein kinase structural domains^[138]^	Clinical approval for the combination treatment of past anthracycline, taxane, and trastuzumab-treated advanced or metastatic HER2-positive breast cancer patients with capecitabine. (2007-03-13)
Neratinib	HER1, HER2, HER4	Prevents autophosphorylation of tyrosine residues on HER1, HER2 and HER4 receptors by covalent binding to cysteine side chains in the ATP-binding pouch of the receptors^[139,140]^	Clinical approval for extended adjuvant therapy following trastuzumab adjuvant therapy in adult individuals with early-stage *HER2*-amplified breast cancer. (2017-07-17)
Margetuximab	HER2’s extracellular subdomain IV near the membrane	Binds to subdomain IV of HER2 and disrupts HER2 signaling, producing antiproliferative effects comparable to trastuzumab and inducing more efficient ADCC^[141]^	Clinically approved for combination with chemotherapy for the treatment of adult patients with metastatic HER2-positive breast cancer who have previously received two or more HER2-targeted therapies.^[141,142]^ (2020-12-16)
Tucatinib	HER2	Targeting HER2’s structural kinase domain^[137]^	Clinical approval for combination with trastuzumab and capecitabine for treatment of adult patients with advanced unresectable or metastatic *HER2*-amplified breast cancer (2020-04-17)
Pyrotinib	HER1, HER2	Targeting the structural domain of receptor tyrosine kinases and comprehensively blocking the downstream signaling pathway of HER family homo-/heterodimers	First global conditional approval in China for the combination with capecitabine in advanced or metastatic HER2-positive breast cancer patients who have previously received prior anthracycline or paclitaxel-based chemotherapy^[143]^ (2018-08-12)
MP0274	HER2	Binding to two distinct sites on HER2 leads to unique pro-apoptotic effects	Clinical phase 1 trials
Trastuzumab-dkst (MYL1401O), trastuzumab – pkrb (CT-P6), trastuzumab – dttb, trastuzumab – qyyp, trastuzumab – anns	Similar to trastuzumab		Clinically approved for the management of HER2-positive breast cancer. trastuzumab-dkst (2017), trastuzumab-pkrb (2018-12), trastuzumab-dttb (2019-01), trastuzumab-qyyp (2019-03), trastuzumab-anns^[28]^(2019-06-13)

ADCC = antibody-dependent cell-mediated cytotoxicity, HER2/ErbB2 = human epidermal growth factor receptor 2.

### 8.2. PI3K inhibitors

Pan-PI3K inhibitors, such as pilaralisib (XL147), buparlisib (BKM120) and pictilisib (GDC-0941), block all class IA PI3Ks,^[144]^ with BKM120 and GDC0941 showing activity in HER2-positive cells and xenograft models to inhibit HER2 expansion proliferation in in vitro models. The inhibitors alpelisib (BYL719) and taselisib (GDC-0032) selectively target the PI3K isoforms p110α, β, γ, or δ. Among them, the inhibitor BYL719, which targets p110α specifically, has demonstrated increased efficacy in various breast cancer cell lines with mutations in *PIK3CA* and HER2 positivity.^[22,144]^

### 8.3. AKT inhibitors

Competitive inhibitors of AKT (afuresertib, capivasertib, ipatasertib, uprosertib) and allosteric inhibitors (MK-2206, TAS-117, ARQ092/miransertib, BAY1125976) are being studied in preclinical and clinical settings.^[22]^ Capivasertib is the world’s first FDA-approved AKT inhibitor that is used in combination with fulvestrant to treat breast cancer patients who meet specific criteria (2023-11-17). Allosteric inhibitors have been used in many early trials in advanced solid tumors, the preliminary efficacy of the MK-2206 and trastuzumab combination in phase I trials for HER2-positive advanced breast and gastroesophageal tumors has been demonstrated through a study on various subtypes of breast cancer.^[145]^

## 9. Mechanisms of drug resistance targeting the HER2-AKT pathway

There are 3 main mechanisms of resistance targeting the HER2–AKT pathway. The first is the effect of alterations on the structure and surroundings of HER2, such as activation by HER2 splice isoforms or mutations. The second is the activation of HER2 downstream signaling pathways, and dysregulation of the PI3K pathway plays an imperative role in the progression of malignant tumors. The third involves the effects of other factors, such as the interaction of HER2 with other membrane receptors (HER3, IGF-1R) and reactivation of AKT by kinases.

### 9.1. Changes in HER2 structure and surroundings

One study demonstrated that *HER2(L755S*) mutations can restrict the effectiveness of trastuzumab and lapatinib treatments in individuals diagnosed with HER2-positive breast cancer^[146]^ and are the most prevalent alterations correlated with lapatinib resistance in previously trastuzumab-treated metastatic breast cancer.^[28,36]^ *HER2 (L755S* and *V777L*) mutations overactivate the HER3/PI3K/AKT/mTOR axis and induce endocrine resistance, conferring anti-estrogen resistance in ER-positive breast cancer.^[147]^

Both Δ16HER2 and p95HER2, representing distinct variants of HER2, are essential contributors to the emergence of resistance against trastuzumab in the process of its development.^[28,115]^ Breast cancers expressing p95HER2 have a worse prognosis and are more likely to develop resistance to trastuzumab.^[84]^ The cyclin D1–cyclin-dependent kinase 4 (CDK4) pathway has been shown to participate in resistance to HER2-targeted treatments, and CDK 4/6 inhibitors can make resistant tumor cells more responsive to HER2 treatment.^[115]^

Upregulated expression levels of the membrane-associated glycoprotein MUC4 masked the trastuzumab-binding epitope of HER2 and hindered the anti-proliferative and antibody-dependent cell-mediated cytotoxicity effects of trastuzumab; in addition, MUC4 expression contributed to T-DM1 resistance.^[148,149]^ Clinically, based on DNA sequencing data from tumor tissue and peripheral blood, it has been observed that breast cancer patients with HER2 amplification and concurrent CDK12 amplification tend to exhibit a lower response to anti-HER2 therapy.^[150]^

### 9.2. Activation of HER2 downstream signaling pathways

The PI3K/AKT pathway is crucially implicated in several malignancies, and factors associated with resistance to HER2-directed drugs are invariably related to PI3K/AKT pathway reactivation.^[151,152]^ Preclinical model data suggest that deletion mutations in PTEN function or dominant activating mutations in PIK3CA may provide additional inputs to the PI3K pathway independent of the HER2/HER3 heterodimer, ultimately leading to resistance to HER2-targeted drugs.^[153–156]^ In addition to mutational activation of the PI3K/AKT pathway, there are also cross-interferences with other pathways that affect the activation of PI3K/AKT pathways. The activities of the PI3K/AKT pathway and the Ras/ERK pathway are regulated by cross-inhibition, pathway cross-activation, and pathway convergence between them. Cross-inhibition refers to the negative regulation of each other’s activities, wherein when one pathway is chemically obstructed, cross-inhibition occurs and effectively activates the alternative pathway. Pathway cross-activation is the cross-activation of PI3K–mTORC1 signaling by the Ras/ERK pathway through the regulation of PI3K, TSC2, and mTORC1.^[21,52]^

A novel *AKT3* mutation was identified in the lesions of HER2-positive breast cancer patients receiving trastuzumab monotherapy. Cell proliferation assays showed that HER2-overexpressing cellsexpressing the *AKT3 R247C* mutant exhibit enhanced mTORC1 activity and increased trastuzumab tolerance, suggesting that *AKT3 R247C* may be involved in acquiring resistance to anti-HER2 therapy.^[22]^ Tamoxifen-resistant human breast cancer cell lines show an increase in the expression and activity of AKT3.^[157]^

### 9.3. Impacts of other factors

Due to the compensatory transition of phosphorylation-dephosphorylation balance in HER3, and the buffering effect of HER3 signaling on incomplete inhibition of HER2 kinase, HER3 and subsequent PI3K/AKT signaling are protected from inhibition by the present HER family TKIs in tumors in vitro and in vivo.^[158]^ The HER3 E928G kinase domain mutations have been demonstrated to improve HER2/HER3 affinity and reduce HER2 binding to neratinib. The coexpression of *HER2* and *HER3* mutations leads to enhanced activation of the PI3K/AKT pathway and resistance towards neratinib.^[28]^

In lapatinib-resistant HER2-positive breast cancer cells, PI3K/AKT and MAPK signaling was maintained despite sustained suppression of the HER2 tyrosine kinase, and the tyrosine phosphorylated proteomes of both sensitive and drug-resistant cells were analyzed by immunoenrichment mass spectrometry, which revealed that the phosphorylation of Src family kinases and presumptive Src substrates in some resistant cell lines was increased. PI3K/AKT signaling was partially blocked in these resistant cells by treatment with Src kinase inhibitors and lapatinib sensitivity was restored. Phosphopeptides of the Src family kinases Yes (Y222 and Y426) were found to be more abundantly expressed in lapatinib-resistant cells by spectral counting, suggesting a possible role for the Src family of kinases in mediating resistance and demonstrating that Phosphorylated Yes(Y222) was mainly detected in resistant cells.^[152]^ Furthermore, activation of SRC itself was associated with trastuzumab resistance.^[115]^

TNFα is a critical factor in the development of trastuzumab resistance in vivo for HER2-positive breast cancer. It exerts a pivotal influence on trastuzumab resistance through the TNFα-NF-κB-MUC4 pathway, conferring resistance against trastuzumab in both HER2-positive breast and gastric cancers. The resistance mechanism relies on the induction of MUC4 expression by TNFα.^[149]^

The human 17q21 region is a gene-rich chromosomal amplification region containing several candidate cancer genes, such as *HER2* and *DARPP32*. It was found that t-DARPP prolonged the half-life of HER2 and could form a protein complex with HER2, interfering with the bonding of trastuzumab to the HER2 receptor, which could effectively inhibit trastuzumab-induced apoptosis and block trastuzumab-induced dephosphorylation of HER2 and AKT proteins to promote drug resistance to trastuzumab.^[159]^

Trastuzumab resistance was found to be associated with reduced levels of p27^kip1^ by creating a trastuzumab-resistant cell line in SKBR3 to study the mechanism of escape from trastuzumab-mediated growth inhibition.^[160]^ Previous studies have shown that elevated levels of IGF-IR signaling and the formation of heterodimers with HER2 contribute to the action of trastuzumab^[161]^ and that the role of IGF-I in mediating resistance to trastuzumab is related to increase the ubiquitination-mediated degradation of p27^kip1^, resulting in a reduction in diminished protein levels.^[162]^ Ligand-induced stimulation of the Met receptor shields cells from trastuzumab-mediated growth suppression by inhibiting p27 induction. Met may also be responsible for trastuzumab resistance mediated by IGF-IR.^[163]^

Activity-enhancing protein kinase A may be involved in trastuzumab resistance via 3 pathways, this encompasses both direct stimulation of EGFR or PI3K, along with indirect activation of AKT through protein phosphatase 1(PP1) blockade.^[164]^ Finally, fatty acid synthase confers resistance to trastuzumab in HER2-positive breast cancer via membrane lipid rafts,^[165]^ polyomavirus enhancer activator 3, and ERα.^[166–169]^

## 10. Summary and future perspectives

The critical oncogenic potential of HER2 overexpression in breast cancer makes HER2 an attractive target for the therapy of targeted anti-tumor drugs. Over time, tremendous advances have been made in the treatment of patients with early- and late-stage HER2-positive breast cancer, but the death of HER2-positive patients remains inevitable due to the emergence of drug resistance.

In this review, we have summarized the gene amplifications and mutations commonly observed in HER2-positive breast cancer cells, these alterations frequently result in abnormal activation of the HER2 downstream PI3K/AKT/mTOR signaling pathway. This activation plays a crucial role in promoting cell proliferation, inhibiting cell death, and causing drug resistance in breast cancer cells. Thus, targeting the HER2/PI3K/AKT pathway shows great potential for therapeutic intervention in HER2-positive breast cancer patients.

Currently, there is increasing emphasis on optimizing treatment regimens for HER2-positive patients through therapy de-escalation or escalation, novel combination therapies are the focus of attention.^[4]^ We focused on summarizing HER2 targeted drugs that have been approved for clinical combination therapy or currently undergoing clinical trials, while also examining the underlying causes of resistance to HER2-targeted treatment. Excluding aberrant activation caused by structural changes in the HER2/AKT pathway itself, changes in the surrounding environment of HER2, cross-talk activation of pathway signaling molecules, and involvement of other proteins can confer resistance to trastuzumab in breast cancer cells. In addition to investigating compounds that may more effectively suppress HER2 signaling, it is crucial to inhibit the complex and redundant interactions between the HER2 network and other signaling pathways in order to minimize drug side effects. For drugs currently approved by the FDA, recent research indicates that combining HER2 targeted therapy with AKT pathway inhibitors (such as PI3Ki, CDK4i/CD6i) in order to completely block the activation of the HER2/AKT pathway can increase treatment options for existing and potential patients, as well as reduce the drug toxicity associated with HER2 directed therapy, thereby further improving patient prognosis.

## Author contributions

**Conceptualization:** Xuesen Li, Xiaoping Wu, Linghui Pan, Zili Gao, Mao Yang, Jinling Li.

**Funding acquisition:** Xuesen Li, Xiaoping Wu.

**Visualization:** Linghui Pan, Jinling Li, Qi Xu.

**Writing – original draft:** Linghui Pan.

**Writing – review & editing:** Xuesen Li, Linghui Pan, Jinling Li, Qi Xu, Zili Gao, Mao Yang.
